# Logistics of an advanced therapy medicinal product during COVID-19 pandemic in Italy: successful delivery of mesenchymal stromal cells in dry ice

**DOI:** 10.1186/s12967-020-02625-0

**Published:** 2020-11-30

**Authors:** Giuseppe Astori, Martina Bernardi, Angela Bozza, Daniela Catanzaro, Katia Chieregato, Anna Merlo, Monica Santimaria, Roberto Barbazza, Giuseppe Amodeo, Rachele Ciccocioppo, Francesca Elice, Marco Ruggeri

**Affiliations:** 1 Advanced Cellular Therapy Laboratory, Haematology Unit, Vicenza Hospital, Contrà S. Francesco, 41, 36100 Vicenza (I), Italy; 2 Consorzio Per la Ricerca Sanitaria’ (CORIS) of the Veneto Region, Padua, Italy; 3 Nuclear Medicine Service, Department of Diagnostics, Vicenza Hospital, Vicenza, Italy; 4 Pharmacy Service, Department of Medical Management, A.O.U.I. Ospedale Maggiore, Verona, Italy; 5 Gastroenterology Unit, Department of Medicine, A.O.U.I. Policlinico G.B. Rossi and University of Verona, Verona, Italy; 6 Haematology Unit, Vicenza Hospital, Vicenza, Italy

**Keywords:** COVID-19, GvHD, Mesenchymal stromal cells, Supply chain

## Abstract

**Background:**

During the coronavirus disease-2019 (COVID-19) pandemic, Italian hospitals faced the most daunting challenges of their recent history, and only essential therapeutic interventions were feasible. From March to April 2020, the Laboratory of Advanced Cellular Therapies (Vicenza, Italy) received requests to treat a patient with severe COVID-19 and a patient with acute graft-*versus*-host disease with umbilical cord-derived mesenchymal stromal cells (UC-MSCs). Access to clinics was restricted due to the risk of contagion. Transport of UC-MSCs in liquid nitrogen was unmanageable, leaving shipment in dry ice as the only option.

**Methods:**

We assessed effects of the transition from liquid nitrogen to dry ice on cell viability; apoptosis; phenotype; proliferation; immunomodulation; and clonogenesis; and validated dry ice-based transport of UC-MSCs to clinics.

**Results:**

Our results showed no differences in cell functionality related to the two storage conditions, and demonstrated the preservation of immunomodulatory and clonogenic potentials in dry ice. UC-MSCs were successfully delivered to points-of-care, enabling favourable clinical outcomes.

**Conclusions:**

This experience underscores the flexibility of a public cell factory in its adaptation of the logistics of an advanced therapy medicinal product during a public health crisis. Alternative supply chains should be evaluated for other cell products to guarantee delivery during catastrophes.

## Background

Advanced therapy medicinal products (ATMPs), including stem cells, are usually stored and cryopreserved in liquid nitrogen (LN_2_) and delivered to points-of-care by authorized couriers using dry vapour (dewar) shippers. This enables the direct transport of ATMPs to points-of-care, where they are thawed and infused at the bedside, primarily because hospital pharmacy services do not have access to LN_2_ freezers. Moreover, vapour-phase shipment requires not only specific logistical resources, but also strict adherence to handling procedures for LN_2_ supplies by trained personnel [1]. During the severe acute respiratory syndrome coronavirus-2 (SARS-CoV-2) pandemic, Italian hospitals faced the most daunting challenges of our recent history. In this context, the capability to provide ATMPs was impeded by the urgent need to prevent viral transmission to trained couriers and other ancillary personnel.

Mesenchymal stromal cells (MSCs) are non-hematopoietic progenitors that can be isolated from several adult and fetal tissues, and can differentiate into three cellular lineages under appropriate stimuli. Their characterization is based upon criteria defined by the International Society for Cell & Gene Therapies [2, 3], which include a common phenotype, even though specific markers have been identified to distinguish MSCs from different sources [4, 5]. In the last decade, both scientists and clinicians have investigated the potent immunomodulatory effects of MSCs on all immune effector cell subpopulations, [6, 7] particularly T-cells [6, 8–10]. Consequently, MSCs are an attractive therapeutic candidate for autoimmune disorders [11] such as acute graft-*versus*-host disease (aGvHD), a complication of bone marrow transplantation that carries high morbidity and mortality [12, 13]. Thus, Dazzi and colleagues demonstrated in an elegant work that MSC death may drive immunosuppression, and that the cytotoxic activity of T lymphocytes and NK cells against MSCs may predict clinical response [14]. Notably, when infused intravenously, the vast majority of MSCs are entrapped in the lungs [15], although neither their localization nor their engraftment to the target organ are required for their efficacy. Another condition for which MSCs have been used is severe coronavirus disease-2019 (COVID-19), in which pathogenesis is triggered by a virus-induced exaggerated immune response (“cytokine storm”) [16] that causes an acute respiratory distress syndrome (ARDS). Indeed, based on the safety and possible efficacy of MSCs in ARDS trials [17, 18], a case series of seven patients suffering from severe COVID-19 pneumonia were treated with single MSC intravenous infusions (1 × 10^6^ cells/kg) at the Beijing YouAn Hospital, and experienced improved clinical outcomes [19]. Numerous clinical trials are ongoing worldwide.

During the Italian pandemic (March to April 2020), the Laboratory of Advanced Cellular Therapies (LTCA, Vicenza, Italy) received requests for serial infusions of umbilical cord-derived mesenchymal stromal cells (UC-MSCs) for a patient with COVID-19-related pneumonia and for a patient with severe intestinal aGvHD. At that time, access to clinics and particularly to COVID-19 units by trained LN_2_ couriers and other ancillary personnel was prohibited due to the high risk of contagion that forced hospitals to execute unprecedented internal reorganizations and restrictive personnel management policies. Transport of UC-MSCs to the clinics in LN2 was unmanageable. The need to ensure the continuity of the therapeutic supply chain prompted us to validate the shipment of cell bags in dry ice as the only possible alternative transport strategy. Dry ice is the solid form of carbon dioxide that sublimates at −78.5 °C at sea level. The validation process comprised both prospective assessments of the effects of the transition from LN_2_ to dry ice on cell function and a concurrent validation during delivery to the bedside. Our successful experience underscored the flexibility of a public cell factory to adapt logistics during a public health crisis, and emphasized need to test additional transport methods to guarantee the transport of other cell products to points-of-care.

## Methods

### Advanced Therapy Medicinal Product production and cryopreservation

When used as therapeutic agents, MSCs are classified as “Advanced Therapy Medicinal Products” (ATMPs) according to European regulation no. 1394/2007. Each ATMP must be produced following Good Manufacturing Practice (GMP) rules in facilities authorized by the national regulatory body. Umbilical cords (UCs) for MSC production were collected during caesarean sections. The mothers provided signed informed consent (Ethics Committee act no. 16/18 of February 3rd, 2018), and their blood was screened according to European Directive 2004/23/EC, for HCV/HBV/HIV1-DNA, anti-HIV 1 + 2 Ab, anti-HCV Ab, HBsAg and anti-*T. pallidum* Ab. Briefly, after collection, UCs were immediately submerged in a decontamination solution containing a cocktail of four antibiotics (BASE 128; Alchimia; Padua, Italy). Then, in the clean room, UCs were first minced into small fragments, seeded in conventional T-flasks in Alpha MEM (Stem Cells Technologies; Canada) added with 5% human-derived platelet lysate (produced by the blood bank of Meyer Hospital; Florence, I) and 2U/mL heparin (Pharmatex Italia; Milan, Italy). Cells were incubated at 37 °C and 5% CO_2_. After 7 days, tissue fragments were removed and medium was substituted. After 13 days, adherent cells were detached by using TrypLe Select (Thermo Fischer; Waltham, MA, USA) and re-seeded in complete medium. On Day 19, adherent cells were detached and expanded in HYPERFlask Cell Culture Vessels (Corning; NY, USA) and on Day 26 cells were harvested, counted and collected in CryoMACS freezing bags (Miltenyi Biotec; Bergisch Gladbach, DE) or in cryotubes (Nalgene; Rochester, NY, USA). Cells were finally frozen to − 140 °C in a cryogenic freezer (Feezal, Air Liquide; Paris, FR) at a cooling rate of − 1 °C/min to − 40 °C followed by a cooling rate of − 5 °C/min to − 140 °C, and finally stored in vapour phase LN_2_. UC-MSCs bags were released after the quality control check that included sterility, mycoplasma and endotoxins quantification, cell count, phenotype, karyotype, cell viability and impurities tests compliant with European Pharmacopeia methods.

### In vitro experimental design

**Fig. 1:**
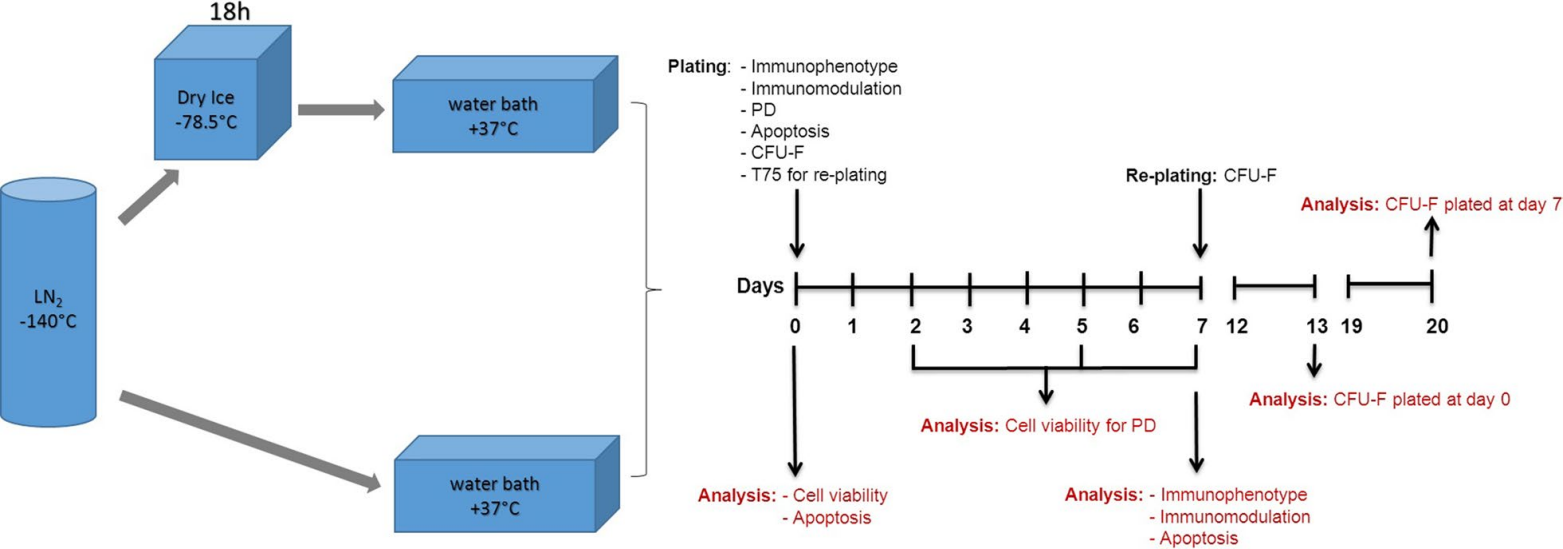
Experimental design. To simulate the different shipment modalities, cells stored in LN2 vapour were thawed directly at 37 °C or after incubation for 18 h in dry ice. Cell viability and apoptosis were determined immediately after thawing. Immunophenotype, immunomodulation, and apoptosis were analysed after one week in culture. Population doubling was determined by counting cells at days 0, 2, 5 and 7. CFU-F potential was determined on cells plated immediately after thawing or plated after one week in culture (n = 3)

To simulate the conditions to which cells would be exposed during the alternative shipment method, cells stored in LN_2_ vapour (n = 3) were incubated for 18 h in dry ice before thawing; as a control, cells stored in LN_2_ were thawed directly at 37 °C. Analyses of immuno-phenotype; immuno-modulation; population doubling (PD); cell viability, apoptosis; and colony forming unit-fibroblast (CFU-F) potential were performed as described in Fig. 1.

### Cell viability, apoptosis and population doubling

To test viability after thawing, cells were harvested and resuspended in trypan blue (Thermo Fischer) at 1:1 ratio (v:v) in medium solution. The number of viable/dead cells was scored by using a Burker hemocytometer. For apoptosis testing, 2 × 10^5^ cells were harvested after thawing and centrifuged at 400 g for 6 min at 8 °C. Cells were then washed with binding buffer and labelled with Annexin V/7-aminoactinomycin D (7-AAD) (Thermo Fischer). After dilution with the binding buffer (1:10 v:v), fluorescence of 2 × 10^4^ cells/sample was detected by using a Cytomics FC500 cytometer and analysed by using EXPO32 software (all by Beckman Coulter; Brea, CA, USA). Cell populations were separated into four subsets: viable cells were negative to both Annexin V and 7-AAD fluorescence; cells in early phase of apoptosis were Annexin V^+^/7-AAD^–^; cells in late phase of apoptosis were Annexin V^+^/7-AAD^+^, while the necrotic cells were Annexin V^−^/7-AAD^+^. Cell viability and apoptosis were determined for both transport conditions (LN_2_ and dry ice) immediately after thawing (Day 0) and after one week in culture (Day 7). In the last condition, the PD was calculated after 2–5-7 days as follows: PD = (Log_10_ Nt – Log_10_ N0)/ Log_10_ 2, where Nt = number of counted cells N0 = number of plated cells (n = 3).

### Immunophenotype

Briefly, 1 × 10^5^ cells were stained with monoclonal anti-human antibodies against CD31-FITC (Clone 5.6E), CD45-ECD (Clone J.33), CD105-PE (Clone 1G2), CD90-FITC (Clone F15-42–1-5), HLA-DR-APC (Clone IMU 357) and 7-amino actinomycin D (7-AAD) (all by Beckman Coulter) and CD34-PE (Clone 8G12), CD73-PC7 (Clone AD2) (by Beckton Dickinson; Franklin Lakes, NJ, USA). Cells were incubated for 15 min at room temperature together with specific antibodies (CD90/CD105/CD45/7AAD; CD31/CD34/CD45/HLA-DR/CD73). After washing, at least 10,000 events were acquired using a FC500 flow cytometer and analysed using Kaluza software (both by Beckman Coulter).

### Immunomodulation

Thawed peripheral blood mononuclear cells (PBMCs) collected from a single healthy donor were suspended in RPMI 1640 (Sigma-Aldrich; St. Louis, MO, USA) supplemented with 10% FBS, 2 mM L-glutamine, 100U/ml penicillin, 0.1 mg/ml streptomycin (all by Sigma-Aldrich) and rested overnight at 37 °C in a humidified atmosphere containing 5% CO_2_. UC-MSCs were seeded in 96-well flat-bottom plates (Corning) at different densities: 4 × 10^4^, 2 × 10^4^ and 1 × 10^4^ cells. To measure cell proliferation, PBMCs were stained with 5 μM 5,6-carboxyfluorescein diacetate succinimidyl ester (CFSE) (CellTrace Cell Proliferation Kit; Invitrogen, Carlsbad, CA, USA) according to manufacturer’s instructions. PBMCs were also stimulated with 0.5 μg/ml of anti-CD3 antibody (clone OKT3, Miltenyi Biotec) and 500 IU/ml of recombinant human interleukin-2 (rh-IL-2, Miltenyi Biotec) for six days before measuring the corresponding decrease in CFSE fluorescence by flow cytometry. Stimulated and unstimulated PBMCs seeded alone were used as controls. CFSE-labeled PBMCs (2 × 10^5^) were then seeded on MSCs monolayers to obtain different MSC:PBMC ratios of 1:5, 1:10 and 1:20. Anti-human CD45-ECD antibody was used to assess proliferation on gated CD45^+^ cells. At least 20,000 events were acquired on a Cytomics FC500 cytometer (Beckman Coulter). PBMC proliferation was quantified as the percentage of cells undergoing at least one cell division (n = 3).

### Colony forming unit-fibroblast assay

UC-MSC clonogenic potential was evaluated by seeding 200 cells in a 100-mm plate (Corning), in duplicate for each condition, immediately after thawing (Day 0) or after 7 days (Day 7) in culture. Cells were incubated at 37 °C and 5% CO_2_ and the medium was changed at Day 7. After 13 days in culture, cells were washed, fixed with 10% formaldheyde and stained with 0.1% Crystal Violet for one hour at room temperature. Only colonies with a minimum of 30 cells were scored (n = 3).

### Validation of ATMP delivery in dry ice

Two concomitant validation runs were performed during the transport of the ATMP from the LTCA in Vicenza to the Intensive Care Unit (ICU) of Ospedale Maggiore in Verona (approximately 60 km distant, corresponding to less than one hour of driving), and from the LTCA to the Haematology Unit (both in Vicenza, approximately 500 m distant, corresponding to ten minutes of walking by using the authorized courier of the Vicenza Hospital. A third run (worst case) was conducted by maintaining the box container at 35 °C in a hood (Heraeus; Hanau, DE) for 24 h in order to “challenge” the system. The goal was to validate the box container and packaging instructions; the quantity of dry ice to be loaded; and the maximum allowable transport time. Packaging was done in compliance with UN3373 (Biological substance, Category B) in UN1845 Dry Ice, IATA label Class 9. The box, constituted of high-density polystyrene foam (27.5 × 25.0 × 22.5 cm and 3.5 cm thick), was loaded with dry ice in pellets (5kg). The dry ice parcel-sized shippers comprised the payload area surrounded by an insulation medium, with the product submerged in dry-ice pellets. The box was filled halfway with dry ice and the bag containing the cells was placed horizontally in contact with a datalogger probe (iLog, Escort Scunthorpe, UK). The box was filled to the brim and closed with the lid sealed with tape. The temperature was recorded every 5 min and the box was delivered at room temperature by using the authorized courier of the Verona Hospital. As acceptance criteria, the change in weight of dry ice at the end of the worst case test could not exceed 10%, and the temperature of the bags could never exceed − 76 °C.

### ATMP administration

The ATMP was supplied as a frozen, sterile, apyrogenic product in bags containing a concentration of 1 to 2 × 10^6^ cells/ml. Cells were thawed by continuous agitation in sterile conditions by submerging the bag with an overpouch in a water bath at 37 °C. Once thawed, the overpouch was removed and cells were diluted 1:1 (50 ml final) in thawing solution consisting of 38% saline; 50% human albumin 20% v/v (10% final); and 12% anticoagulant Citrate Dextrose Solution (Terumo; Rome, I). The bags were connected to infusion sets, delivered immediately to the clinics, and infused intravenously to patients in 30 min. The treatment consisted of two infusions of 1.1 × 10^6^ UC-MSCs/kg body weight one week apart for the COVID-19 patient, and three infusions of 1.5 × 10^6^ UC-MSCs/kg body weight one week apart for the aGvHD patient.

### Statistical analysis

The two-tailed Student’s *t*-test was used to analyse statistical differences between groups. P-values < 0.05 were considered statistically significant. All experiments were performed in triplicate. Data are expressed as mean ± standard deviation (SD). All statistical analyses were performed by using GraphPad Prism software (San Diego, CA, USA).

## Results

### Cell viability, recovery yield and population doubling rates

**Fig. 2:**
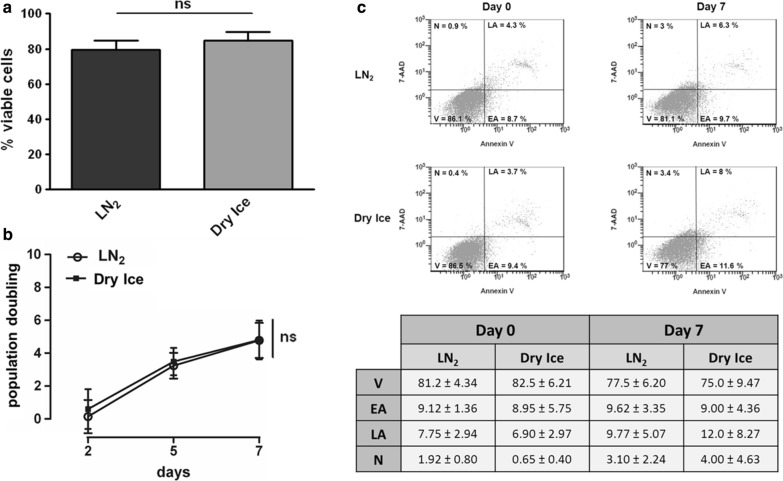
Analyses of cell viability, apoptosis rates and population doubling. **a** Histograms of the percentage of viable cells after thawing, measured with trypan blue exclusion assay. **b** Population doubling (PD) calculated at Days 2, 5 and 7 of culture post-thawing. **c** Representative cytofluorimetric plots of apoptosis analyses and relative results expressed as percentages. Viability (V), early apoptosis (EA), late apoptosis (LA), and necrosis (N) of cells stored in LN_2_ and dry ice were analysed immediately after cell thawing (Day 0) or after one week in culture (Day 7). Data are expressed as mean ± SD of three independent experiments. Statistical analysis was performed with GraphPad Prism software and Student’s *t*-test did not evidence any statistical difference

There were no significant differences between cells stored in LN_2_ and dry ice in cell viability, early/late apoptosis, and necrosis immediately after thawing. Indeed, the percentage of viable cells was 81.2 ± 4.3 and 82.5 ± 6.2 respectively. Recovery yield was 101.70 ± 6.51 for cells stored in LN_2_ and 102.62 ± 2.10 for cells stored in dry ice. Similar results were obtained after one week in culture. PD of cells stored in the two conditions showed no statistical differences (Fig. 2).

### Immunophenotyping

UC-MSCs transferred from LN_2_ or dry ice to 37 °C did not show significant differences in the expression of tested antigen markers. Antigen expression was ≥ 99% for CD105, CD90, CD73 and ≤ 2% for CD45, CD31, CD34 and HLA-DR. Viability was ≥ 90% in both cases (Table 1).

**Table 1:** Analysis of immunophenotype

%	NL_2_				Dry ice			
	Batch 1	Batch 2	Batch 3	Mean ± SD	Batch 1	Batch 2	Batch 3	Mean ± SD
7AAD−	95.5	95.2	95.3	95.3 ± 0.2	95.4	93.1	96.6	95.0 ± 1.8
CD105+	99.9	99.7	99.9	99.8 ± 0.1	99.7	99.8	99.8	99.8 ± 0.1
CD90+	99.7	99.7	99.8	99.7 ± 0.1	99.8	99.7	99.6	99.7 ± 0.1
CD73+	100.0	100.0	100.0	100.0 ± 0.0	100.0	100.0	100.0	100.0 ≤ 0.0
CD45+	≤ 2	≤ 2	≤ 2	≤ 2	≤ 2	≤ 2	≤ 2	≤ 2
CD31+	≤ 2	≤ 2	≤ 2	≤ 2	≤ 2	≤ 2	≤ 2	≤ 2
CD34+	≤ 2	≤ 2	≤ 2	≤ 2	≤ 2	≤ 2	≤ 2	≤ 2
HLA DR+	≤ 2	≤ 2	≤ 2	≤ 2	≤ 2	≤ 2	≤ 2	≤ 2

Antigen expression of UC-MSCs transferred from LN_2_ to 37 °C (left) and from dry ice to 37 °C (right). No significant differences were demonstrated

### Immunomodulation

**Fig. 3:**
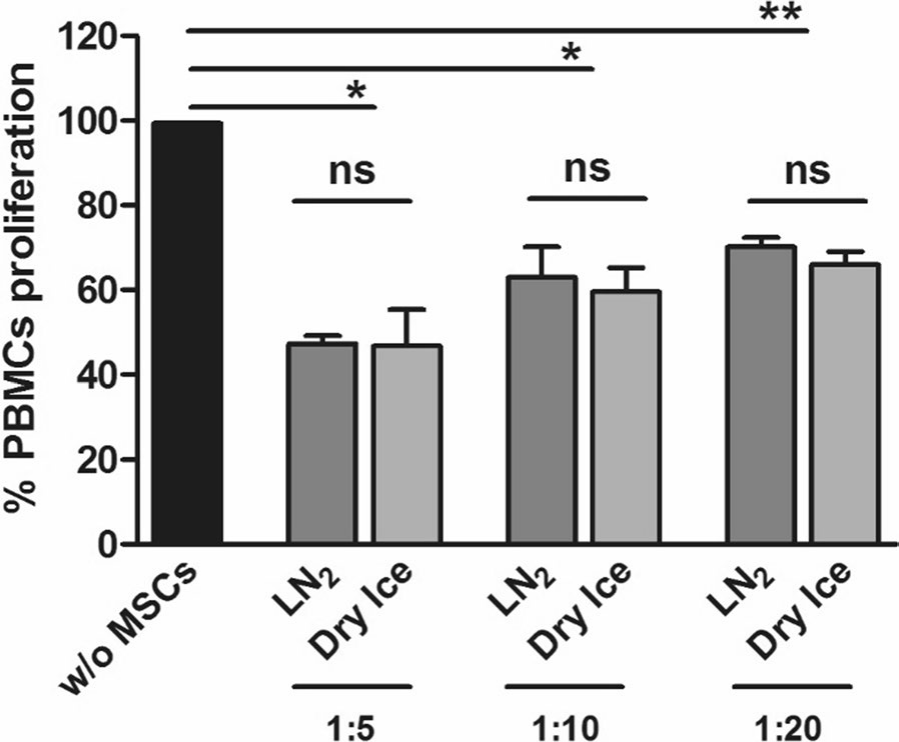
UC-MSCs immunomodulation properties. The anti-proliferative activity of UC-MSCs was analysed by flow cytometry after PBMC labelling with CFSE. Statistical significance is relative to the proliferation of activated lymphocytes in the absence of MSCs (n = 3) (*p < 0.05; ** p < 0.01)

The preservation of immunomodulatory activity after thawing is fundamental to assure the in vitro potency of cells and their clinical efficacy. UC-MSCs stored in dry ice for 18 h before thawing maintained similar antiproliferative activity vs. activated PBMCs in comparison to UC-MSCs thawed directly from LN_2_. Ratio-dependent decrease of PBMC proliferation was maintained in both storage conditions (Fig. 3).

### CFU-F

**Fig. 4:**
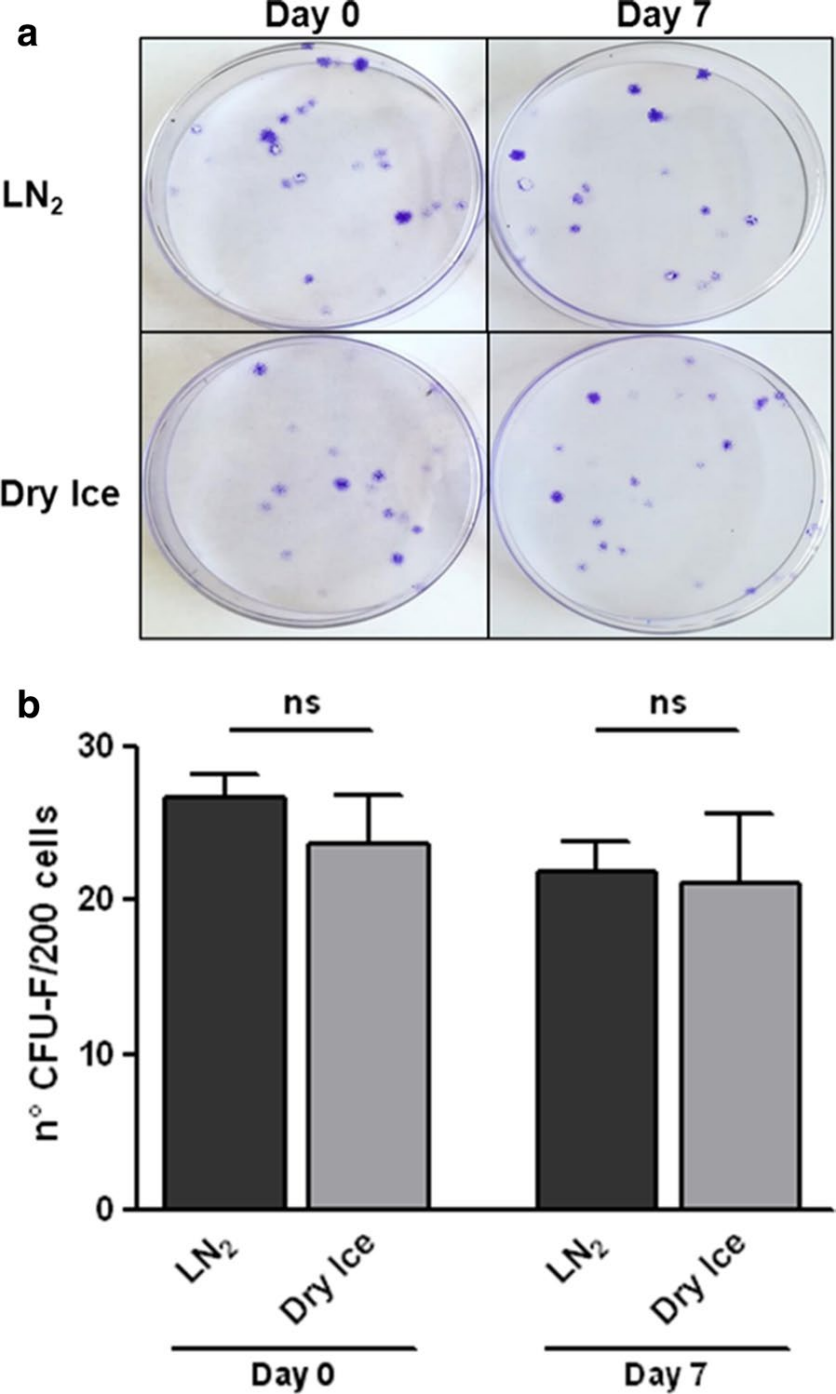
UC-MSC clonogenic potential. **a** Representative images of CFU-F stained with crystal violet. **b** Histograms showing the absolute number of CFU-F obtained from cells thawed after storage in dry ice compared to control cells, thawed after storage in LN_2_. No significant differences were detected

Thawed UC-MSCs maintained their ability to form colonies in culture. Clonogenesis remained identical in both storage conditions (Fig. 4).

### Validation of ATMP transport in dry ice

During ATMP delivery, the temperature of cells always remained below − 78 °C (range from − 80.0 to − 79.0) as confirmed by datalogger records (data not shown). During the “worst case” test, the transport box maintained a stable temperature, even during a 24-h exposure to an external temperature of 35 °C. Dry ice weight loss was limited (30%) (Table 2).

**Table 2:** Results of ATMP transport validation in dry ice

Delivery date	Temperature (external)	Delivery time	Temperature (start)	Temperature (arrival)	Dry ice weight loss
07.04.2020	15 °C	2 h 25 min	− 86.1 °C	− 79.1 °C	2%
14.04.2020	20 °C	1 h 35 min	− 80.3 °C	− 78.9 °C	1%
05.05.2020	35 °C	24 h	− 80.6 °C	− 80.3 °C	30%

Two concurrent and one prospective runs were performed. The temperature of the ATMP always remained below − 76 °C. The maximum delivery time was 24 h

### Advanced Therapy Medicinal Product administration

We treated a patient with respiratory failure due to COVID-19 pneumonia admitted to the ICU of the Ospedale Maggiore (Verona, Italy) and a patient with grade IV steroid-refractory intestinal aGvHD admitted to the Haematology Unit of the San Bortolo Hospital (Vicenza, Italy) with serial intravenous infusions of UC-MSCs under the Hospital Exemption rule (art. 28 of the Regulation (EC) No. 1394/2007). The dosage of UC-MSCs for each infusion was administered using 2 or 3 bags according to the patient’s body weight. During the administration of the content of the first bag, the other bag/s were kept in dry ice. In the absence infusion-related side effects, they were thawed immediately after the completion of the previous bag. No immediate or late adverse events were observed during the four weeks of follow-up in both cases. Moreover, the COVID-19 patient exhibited improved values of numerous laboratory parameters, including biomarkers of inflammation (C-reactive protein from 389 to 24 mg/L; normal value: < 5); renal function (creatinine from 2.27 to 0.56 mg/dL; normal value: 0,59–1,29); coagulation (D-dimer from 3.83 to 0.82 mg/mL; normal value: < 0,25); respiratory function (Pa0_2_/FiO_2_ from 86 to 234; normal value: > 300); and lymphopenia (from 0.6 to 1.48 × 10^9^/L; normal value: 1,2–4.0) following UC-MSC treatment (Day 10) (Ciccocioppo et al., submitted for publication).

The aGVHD patient experienced clinical improvement, with decreased frequency and volume of diarrhoea, reductions of steroid and analgesic dosages; and improvement of cytopenia (one month after the first UC-MSC infusion, leukocyte count increased from 1.4 × 10^9^/L to 3.8 × 10^9^/L, and platelet count rose from 25 × 10^9^/L to 75 × 10^9^/L). This experience underscores the preservation of both the safety and efficacy of a dry ice-shipped cellular product.

## Discussion

The pharmaceutical supply chain of ATMPs faces multiple logistic hurdles compared with conventional drug product transport. Although ATMPs can be shipped as either fresh or cryopreserved products, the possibility that minimal temperature changes may reduce cell potency and safety introduces logistical challenges. Biochemical processes are usually temperature-dependent; even at a range between − 20 °C and− 80 °C, biochemical reactions still occur that result in the accumulation of cytotoxic intermediate compounds such as free radicals, anaerobic metabolism by-products, and waste products that cannot be removed due to the suppression of cellular salvage pathways [20]. Consequently, for successful long-term storage, cells must be frozen to temperatures below the glass transition of the cell cryprotectant mixture (approximately − 120 °C) so that no mobile water fraction persists in stored samples [1]. This is primarily why cells should be stored in LN_2_ in the long term. Consequently, clinical-grade ATMPs are usually delivered in LN_2_ in dry shippers, in which liquid is entrapped in an absorbent material. However, LN_2_ is classified as a hazardous gas due to the risk of suffocation. LN_2_ volume expands 695 times during vaporization, and has no warning properties such as odour or colour. LN_2_ vapours can rapidly freeze skin tissue and ocular fluids, resulting in cold burns, frostbite, and permanent eye injury, even upon brief exposures. If a sufficient quantity is vaporized and the fraction of inhaled oxygen decreases below 19.5%, there is a risk of hypoxia that may cause unconsciousness or death in extreme cases [21]. To prevent asphyxiation, dry shippers should be loaded by trained personnel, and once received at clinics should be manipulated in dedicated rooms.

Historically, cell line shipments to labs have been conducted in dry ice in insulated boxes at approximately − 80 °C. Thermal transport boxes maintain temperatures for time durations related to the quality of the insulation and the quantity of loaded dry ice. Although dry ice is not classified as a hazardous material, prolonged exposures can cause severe skin burns. Because it sublimates into large volumes of carbon dioxide gas that could pose a risk of hypercapnia, thermal boxes containing dry ice should be manipulated in a ventilated environment. Major advantages of dry ice are that it is safer than LN_2_, and that disposable thermal boxes are used. This is not the case for dry shippers; therefore, the shipment in LN_2_ must be performed by a dedicated qualified company.

The advent of the chimeric antigen receptor T-cell (CAR)-T era has placed pharmaceutical companies in a global competition. To facilitate commercial-scale production, “Big Pharma” have centralized CAR-T cell manufacturing in large facilities that are often distant from points-of-care. Although this logistic choice has some advantages, the COVID-19 pandemic has highlighted several systemic weaknesses. Border closures and logistical difficulties that confound patient care and the management of ATMPs have forced clinicians to substantially revise CAR-T therapeutic programs [22]. This pandemic is teaching us that the production and delivery strategies of cell therapy products should be planned proactively to ensure therapeutic continuity even in the case of further closures of national borders.

In April 2020, during the epidemic peak of COVID-19 in Italy, our laboratory faced the challenge of delivering cryopreserved cell product for therapeutic use in a hospital located far from the production site. At that time, transport of UC-MSCs to the clinic in LN_2_ was unmanageable. The only alternative was the transport of the ATMP in dry ice. We had no data regarding the potential effects that cell transitions from LN_2_ to dry ice and then to 37 °C could have on cell efficacy and safety. The literature concerning the effects of temperature fluctuations of frozen product during sample transfer from LN_2_ to dry ice was limited [23–26], so we conducted an internal validation process. Chabot et al. showed that warming of UC-MSCs from LN_2_ to approximately − 80 °C does not have a major impact on MSCs, while internal vial temperatures higher than − 40 °C significantly impair their antiproliferative effects vs. T-cells, and degrade membranous and cytoskeletal integrity [23]. Furthermore, the consequences of thermal variations during cell storage have been the object of several studies conducted with frozen PBMCs [24] and placental-derived MSCs [25]. In these experiments, designed to simulate events in busy bio-repositories, cells were exposed to multiple temperature cycles between LN_2_ gas phase and temperatures as high as − 60 °C. As expected, reductions of both recovery rate and functionality were detectable compared to stable gas phase storage, however, only when the number of cycles exceeded 20 and/or the peak temperature was higher than − 100 °C, thus indicating that cell damage develops only after several temperature transition cycles. Recently, Mareschi et al. compared the post-thawing viability of cytokine-induced killer cells stored in dry ice for 24 or 48 h or in LN_2_ vapours [26]. Because cell viability between the two groups was comparable (acceptance criteria was 15%), the authors concluded that these cells could be shipped safely in dry ice [26].

Under Good Clinical Practice regulations, shipping conditions must also be validated to ensure that the temperature of the product is kept constant during transportation, and that the quantity of refrigerant is sufficient. We have validated the shipping conditions in dry ice from a GMP facility to points-of-care, showing that when using a shipping box containing 5 kg of dry ice, its weight loss was limited (30%) while maintained in very unfavourable conditions at 35 °C for 24 h. Consequently, the choice of in vitro potency assays should take several factors into consideration. First, the trypan blue exclusion text based on membrane integrity is frequently used for the evaluation of post-thaw cell viability, however, this test has several limitations because membrane fluidity that is present immediately after cryopreservation may lead to false-positive or false-negative results. This phenomenon is described as “cryopreservation-induced delayed-onset cell death” [27]. It is therefore essential to select potency assays that verify long-term cell functionality. In addition to immediate testing for cell viability; apoptotic rate; and phenotype; we also evaluated cell proliferation; immunomodulation; and clonogenic capacity. Our results allowed us to conclude that in vitro there are no significant differences between the cells transferred from LN_2_ to 37 °C water bath and those transferred from LN_2_ to dry ice and then to 37 °C water bath in cell viability; apoptosis; phenotype; immunomodulation; and PD. The CFU-F assay has been used historically to evaluate the efficiency with which UC-MSCs form colonies, and it remains a fundamental assay to ascertain the quality of cell preparations. In summary, our data allow us to conclude that there are no differences between the cells stored in LN_2_ and those kept in dry ice for 18 h. Moreover, in the absence of established laboratory parameters to confirm the clinical efficacy of UC-MSCs in COVID-related pneumonia and aGVHD, we have reported favourable clinical outcomes of the two treated patients. Nonetheless, this experience may be considered a preliminary confirmation of the clinical safety and efficacy of this ATMP delivered by a novel shipping method. Conclusive clinical data on the efficacy and safety of dry ice shipped UC-MSCs in other diseases should be obtained in controlled clinical trials.

## Conclusions

In conclusion, UC-MSCs may be shipped safely in dry ice for a limited time duration without causing functional impairment. However, it should be emphasized that different cell types could react differently to a given cryopreservation protocol. Differences in physical and biological characteristics such as membrane permeability and surface-to-volume ratio could produce varying responses to cryopreservation processes, leading to differences in viability after subsequent thawing [28]. Therefore, it is necessary to optimise the cryopreservation, transportation, and thawing protocols for specific cell types of interest. This experience also underscores the fundamental role of a public GMP facility in proximity to points-of-care, not only to reduce transit distances, but primarily for the flexibility to react quickly in critical situations, such as the restrictions imposed during the COVID-19 lockdown.

## Data Availability

The dataset used and/or analysed during the current study are available from the corresponding author on reasonable request.

## Abbreviations

ATMPs: Advanced Therapy Medicinal Products
LN2: Liquid nitrogen
SARS-CoV-2: Severe acute respiratory syndrome coronavirus-2
MSCs: Mesenchymal stromal cells
aGVHD: Acute graft-versus-host disease
COVID-19: Coronavirus disease-2019
ARDS: Acute respiratory distress syndrome
LTCA: Laboratory of Advanced Cellular Therapies
UC-MSCs: Umbilical cord-derived mesenchymal stromal cells
CFU-F: Colony forming unit-fibroblast
UCs: Umbilical cords
GMP: Good Manufacturing Practice
7-AAD: 7-Aminoactinomycin D
CFSE: 5,6-Carboxyfluorescein diacetate succinimidyl ester
ICU: Intensive Care Unit
SD: Standard deviation
